# Räumliche Ausbreitung von COVID-19 durch interregionale Verflechtungen

**DOI:** 10.1007/s10273-020-2674-7

**Published:** 2020-07-06

**Authors:** Andreas Mense, Claus Michelsen

**Affiliations:** 1 FB Wirtschafts- und Sozialwissenschaften, Friedrich-Alexander-Universität Erlangen-Nürnberg, Findelgasse 7/9, 90402 Nürnberg, Deutschland; 2 Konjunkturpolitik, DIW Berlin, Mohrenstr. 58, 10117 Berlin, Deutschland

**Keywords:** I12, I18

## Abstract

Das Coronavirus hat die Weltwirtschaft in eine Krise gestürzt. Seit Anfang März 2020 wurden in Deutschland Kontakt- und Ausgangsbeschränkungen sowie Verbote der Geschäftstätigkeit verhängt, die Schul- und Kitabetreuung ausgesetzt und strenge Hygienevorgaben erlassen. Daraufhin hat sich die Zahl der gemeldeten Neuinfektionen deutlich reduziert. Zudem ist das Gesundheitssystem Deutschlands nicht überlastet. Nicht zuletzt deshalb wird mittlerweile eine intensive Debatte über Lockerungen geführt, die zusätzliche wirtschaftliche Aktivität zulassen würden. Vor allem Pendlerverflechtungen hatten einen großen Anteil an der Ausbreitung von COVID-19 in Deutschland. Schlechte Witterung und eine hohe Bevölkerungsdichte waren weitere Treiber des Infektionsgeschehens.

## Abstract (en)

The coronavirus has plunged the global economy into crisis. Since the beginning of March, contact and exit restrictions and bans on business activities have been imposed in Germany, schools have been closed, child care has been suspended, and strict hygiene regulations have been issued. In the meantime, the number of reported new infections has been significantly reduced and the German healthcare system does not appear to be overburdened to date. This is one of the reasons why there is now an intensive debate about easing the regulations, which would allow additional economic activity. The article also examines commuter links and the role they have played in the spread of COVID-19 in Germany as well as bad weather conditions and a high population density.

Nach nun über acht Wochen weitreichender Einschränkungen in Deutschland liegen mehr und mehr Daten über das Verbreitungsgeschehen des Coronavirus vor, die Aufschluss darüber geben können, welche Faktoren die Verbreitung begünstigt haben. Die Infektionszahlen sind seit Mitte März 2020 erheblich gesunken, sowohl in absoluter Größe als auch in Relation zu den vorherigen Infektionen, einer vereinfacht berechneten Nettoreproduktionsrate. Diese gibt an, wie viele Personen durchschnittlich durch eine in der Vergangenheit erkrankte Person angesteckt wurden. Erste Auswertungen auf Länderebene deuten an, dass vor allem die Schließungen von Kinderbetreuungs- und Bildungseinrichtungen eine Verlangsamung herbeigeführt haben. Eindeutig belegt werden kann diese Hypothese allerdings nicht (Hartl und Weber, 2020).

* Replikationscode für die Schätzungen und Grafiken kann unter http://andreas-mense.de/covid19 heruntergeladen werden.

**Dr. Andreas Mense** ist wissenschaftliche Mitarbeiter am Lehrstuhl für Volkswirtschaftslehre, insbesondere Sozialpolitik an der Friedrich-Alexander-Universität Erlangen-Nürnberg.

**Dr. Claus Michelsen** leitet die Abteilung Konjunkturpolitik am Deutschen Institut für Wirtschaftsforschung in Berlin (DIW Berlin).

So zeigen sich räumlich auch innerhalb der Bundesländer deutlich verschiedene Muster der Verbreitung, was auf weitere wesentliche Einflüsse hinweist. Ihren Ausgangspunkt nahm die Corona-Epidemie Ende Januar in Bayern, als die ersten Fälle nachgewiesen wurden. Bis Ende Februar war das Infektionsgeschehen in Deutschland noch auf wenige Kreise beschränkt. Innerhalb von nur zwei Wochen wurden Infektionen in allen Landesteilen nachgewiesen, allerdings mit deutlich unterschiedlicher Intensität.

In der frühen Phase des Epidemiegeschehens, der 10. Kalenderwoche, waren es vor allem die Ballungsräume des Ruhrgebiets, das Rhein-Main-Gebiet, der Raum Stuttgart oder der Raum München, in denen vermehrte Infektionen gemeldet wurden, ehe die Epidemie nahezu alle Kreise erfasste. Allerdings gab es auch in der späteren Phase ein Gefälle zwischen Stadt und Land, was zunächst auf einen Zusammenhang des Infektionsgeschehens mit der Bevölkerungsdichte hindeutet. Gleichzeitig deuten die räumlichen Muster an, dass es nicht allein die Städte sind, in denen das Virus auftritt. Es verbreitet sich vielmehr auch im Umland der Städte, den klassischen Pendlereinzugsgebieten.

## Empirische Untersuchung möglich

Ausgehend von dieser Beobachtung kann auf Grundlage der Informationen über das Infektionsgeschehen untersucht werden, welche Faktoren die Verbreitung des Coronavirus beeinflusst haben. Als Grundlage dienen die offiziell veröffentlichten Zahlen zum Infektionsgeschehen durch das Robert Koch-Institut (RKI), die für alle Kreise in Deutschland täglich ausgewiesen werden. Naheliegend ist, dass die Wahrscheinlichkeit einer Infektion mit der Wahrscheinlichkeit des Kontakts zu bereits infizierten

Abbildung 1
**Räumliches Infektionsgeschehen im Zeitablauf**

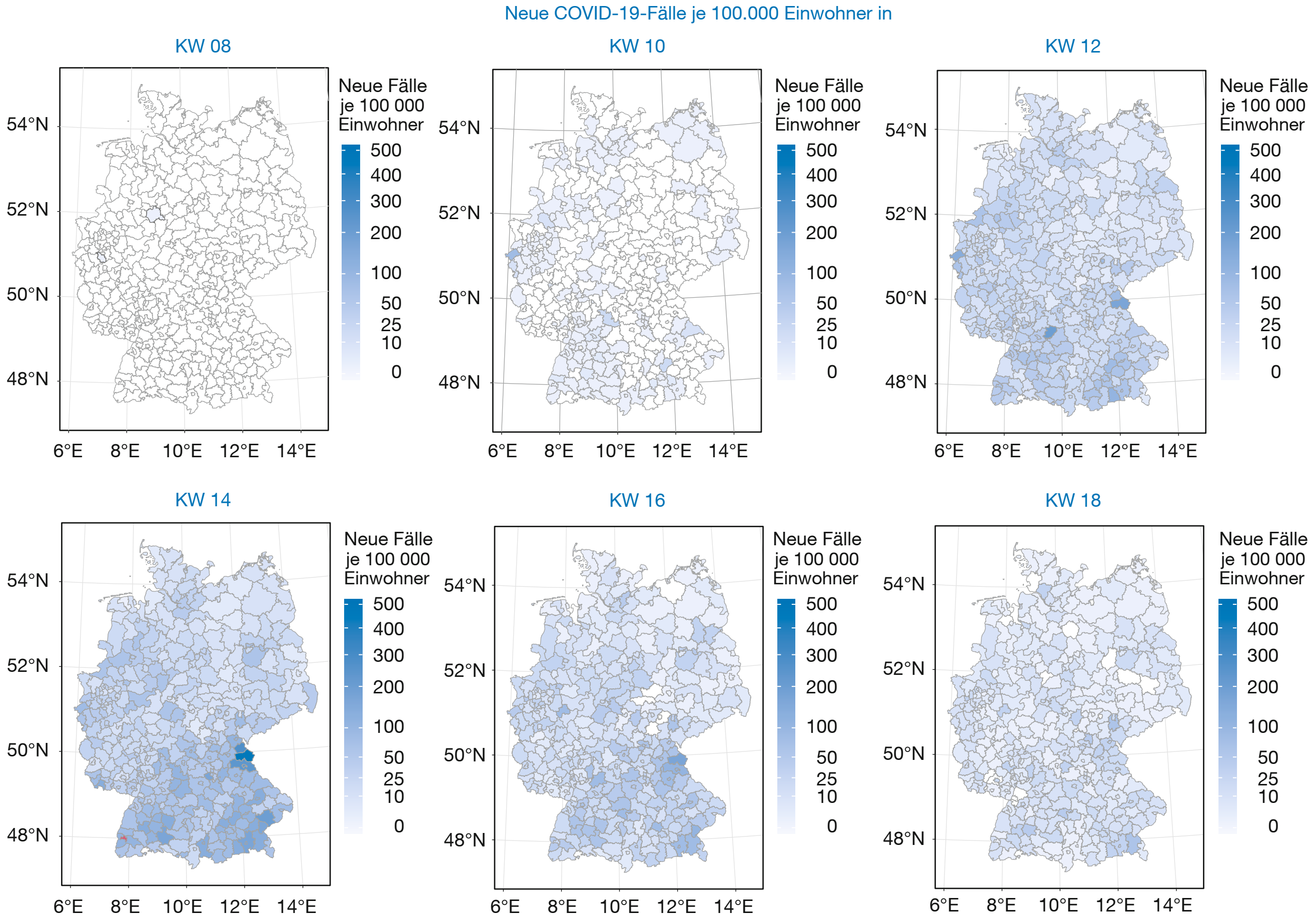

Quellen: Robert Koch-Institut; Bundesamt für Kartographie und Geodäsie; eigene Berechnungen.

Personen steigt. Das Grundmodell geht von der Annahme aus, dass jede in Woche t mit COVID-19 positiv getestete Person eine gewissen Zahl an Personen ansteckt, die dann in der Folgewoche t + 1 als positiv getestet registriert werden. Dabei wird die Zahl der Infektionen innerhalb eines Kreises je Woche betrachtet (vgl. Abbildung 1). Dafür spricht einerseits, dass das Ansteckungsrisiko in den ersten Tagen der Infektion am höchsten ist (an der Heiden und Hamouda, 2020), weswegen ein Großteil des Gesamteffekts innerhalb einer Woche erfasst sein dürfte. Andererseits ergeben sich aus den Test- und Meldeverfahren Wochentagseffekte, die durch die Aggregation verschwinden.

Das statistische Modell besteht aus zwei Komponenten. Zum einen können infektiöse Personen andere Einwohner des gleichen Kreises anstecken. Zum anderen können diese Personen durch Bewegungen über die Kreisgrenzen auch zur Verbreitung des Virus in der Fläche beitragen. Diese beiden Übertragungsmöglichkeiten soll das Modell abbilden:

$$Neue\ Fälle_{i,t} = \psi_i + \phi_t + \beta \times Neue\ Fälle_{i,t-1} + \delta \times \sum_{j \neq i} w_{ij} \times Neue\ Fälle_{j,t-1} + Fehlerterm_{i,t}. \qquad (1)$$

Wenn keine Übertragungen über Kreisgrenzen stattfänden, entspräche $\beta$ in etwa der effektiven Reproduktionszahl. Die Terme $\psi_i$ und $\phi_t$ sind kreis- und wochenspezifische Effekte, die die Schätzungen um die Einflüsse von Unterschieden im Testverhalten zwischen den Kreisen und bundesweiter Änderungen von Test- und Meldeverfahren im Zeitverlauf bereinigen. Es dürfte für den Einfluss anderer Kreise auf das Infektionsgeschehen eine Rolle spielen, wie intensiv Regionen untereinander im Austausch stehen. Enge Pendlerverflechtungen erhöhen die Möglichkeit einer interregionalen Infektionsverbreitung (Adda, 2016). Berufspendler könnten so wesentlich zur Verbreitung des Virus in der Fläche beigetragen haben. Außerdem könnten Pendlerverflechtungen zwischen Kreisen und kreisfreien Städten besonders in den wirtschaftlichen Zentren zu einer Beschleunigung der Dynamik geführt haben. So können mit COVID-19 infizierte Pendler das Virus beispielsweise vom Arbeitsort an den Wohnort bringen, wo sich andere Pendler anstecken, die das Virus wiederum zurück an den Arbeitsort tragen. In diesem Sinne wirken die wirtschaftlichen Zentren als Inkubatoren – weniger, weil in den dicht bevölkerten Städten die Einhaltung der Abstandsregeln schwerer möglich ist als vielmehr aufgrund der engmaschigen Verbindungen zu einer Vielzahl anderer Orte.

Um diese theoretischen Überlegungen empirisch zu prüfen, werden Daten der Bundesagentur für Arbeit zu Ein- und Auspendlern in sozialversicherungspflichtiger Beschäftigung für das Jahr 2019 genutzt. Für je zwei Kreise $i$ und $j$ wird ein Gewicht $w_{ij}$ berechnet:

$$w_{ij} = \frac{Einpendler_{ij} + Auspendler_{ij}}{Einwohner_j} \qquad (2)$$

Weiter wird angenommen, dass sich der Einfluss von Infektionen in einem Kreis $j$ in Woche $t$ - 1 auf die Neuinfektionen in Kreis $i$ in der aktuellen Woche $t$ als $\delta$ x $w_{ij}$ x $Neuinfektionen_{j,t\text{-}1}$ beschreiben lässt. Die theoretische Idee dabei ist, dass die Zahl der Neuinfektionen pro Einwohner in etwa der Wahrscheinlichkeit entspricht, bei einem zufälligen Kontakt mit einer Person aus Kreis $j$ auf eine infektiöse Person zu treffen. Die Summe aus Ein- und Auspendlern gibt die Zahl an Personen an, die sich regelmäßig im jeweils anderen Kreis aufhalten. Der Gesamteffekt der Pendlerverflechtungen auf Kreis $i$ ergibt sich aus der mit den $w_{ij}$ gewichteten Summe der Neuinfektionen in der Vorwoche in allen anderen Kreisen $j$.[1]

In einem zweiten Schritt wird das Modell sukzessive erweitert, um zu untersuchen, in welchen Kreisen die Ausbreitung des Virus eine besondere Dynamik entfalten konnte. Die Zahl der Neuinfektionen in einer Region dürfte sich umso schneller vollziehen, je höher die Bevölkerungsdichte ist. Weiter wurden in sechs Bundesländern – Bayern, Berlin, Brandenburg, Saarland, Sachsen und Sachsen-Anhalt – neben Kontakt- auch Ausgangsbeschränkungen erlassen, was mit einem erhöhten Infektionsgeschehen begründet wurde.

Zudem ist z. B. aus der Literatur zur Wahlforschung bekannt, dass Regenwetter zu geringerer Wahlbeteiligung führt und Bevölkerungsbewegungen insgesamt reduziert (Persson et al., 2014). Es ist anzunehmen, dass bei schlechter Witterung relativ weniger Kontakte im Freien und mehr Kontakte in geschlossenen Räumen stattfinden als sonst. Aus einer höheren Gesamtzahl an Kontakten könnte eine Beschleunigung des Infektionsgeschehens resultieren, sofern Kontakte im Freien dazu einen wesentlichen Beitrag leisten. Andererseits könnten Infektionsketten auch aus einer erhöhten Zahl an zwischenmenschlichen Kontakten in geschlossenen Räumen begünstigt werden. Um die empirische Relevanz der beiden möglichen Übertragungskanäle zu vergleichen, wird ein Interaktionsterm der Zahl der Regentage[2] in der Vorwoche mit der Zahl der Neuinfektionen in der Vorwoche in die Schätzung aufgenommen. Dieser sollte positiv sein, wenn eine höhere Zahl an Kontakten in geschlossenen Räumen die Infektionsgefahr wesentlich stärker erhöht als eine vergleichbare Zahl an Kontakten im Freien.

Das Modell wurde separat für die Kalenderwochen 10 bis 12 und 14 bis 19 geschätzt. Die Kontaktbeschränkungen wurden gegen Ende der Woche 12 erlassen. Wir sparen die Kalenderwoche 13 aus, da die Effekte der Beschränkungen erst mit Verzögerung in den Daten sichtbar sind. Zusätzlich zum oben genannten Modell schätzen wir das Modell separat für jede Woche, um den zeitlichen Verlauf der verschiedenen Dynamiken bildlich darstellen zu können.

### Bevölkerungsdichte und schlechte Witterung wirken sich positiv auf die Verbreitung des Virus aus

Die Ergebnisse zeigen, dass in den Kalenderwochen 10 bis 12 jeder Infizierte im Schnitt etwa 1½ Personen innerhalb des Kreises des eigenen Wohnorts ansteckte (vgl. Tabelle 1, Teil A). Ein großer Teil der Dynamik resultierte jedoch aus der Zahl der Neuinfizierten in Kreisen, die durch Pendlerbeziehungen miteinander verknüpft sind (Modell 1). Nimmt man einen Interaktionsterm mit der Bevölkerungsdichte ins Modell auf (2), so zeigt sich, dass die Dynamik innerhalb von Kreisen mit hoher Bevölkerungsdichte etwa doppelt so stark war wie innerhalb von Kreisen mit niedriger Bevölkerungsdichte. Je höher die Einwohnerzahl/$km^2$, desto höher ist die Ansteckungswahrscheinlichkeit. Die lässt sich mit einer höheren durchschnittlichen Kontaktwahrscheinlichkeit in dichteren Regionen erklären: Je mehr Menschen auf begrenztem Raum leben, desto wahrscheinlicher ist es, dass physische Kontakte zwischen Infizierten und Nichtinfizierten stattfinden und dass sich damit die Verbreitung des Virus beschleunigt. In Bundesländern, die neben Kontakt- auch Ausgangssperren verhängten, war die Dynamik ebenfalls deutlich erhöht (Modell 3). Dies zeigt, dass es für

1 Neben Berufspendlern können auch andere Kontakte über Kreisgrenzen relevant sein. Insofern kann nicht zweifelsfrei geschlossen werden, dass die gemessenen Effekte rein auf die Aktivitäten von Berufspendlern zurückzuführen sind. Als Alternative betrachten wir in einem analogen Ansatz Gewichte $w^*_{ij}$, die den Wert 1 annehmen, wenn die Kreise $i$ und $j$ aneinander angrenzen, und die für alle anderen Kreise gleich 0 sind. Diese Gewichtung könnte für Freizeitkontakte relevanter sein, sofern Freizeitaktivitäten in der Regel in der näheren Umgebung des Wohnortes stattfinden. Die so gebildete Variable hat als zusätzlicher erklärender Faktor keinen Einfluss auf die Zahl der Neuinfektionen und beeinflusst die Ergebnisse auch anderweitig nicht.

2 Die Regentage wurden über die Regnie-Grid-Daten des Deutschen Wetterdienstes auf Kreisebene und Woche aggregiert. Gitterzellen unter 10 mm Niederschlag/$m^2$ wurden als Tage ohne Niederschlag gewertet.

Tabelle 1

**Einflussfaktoren der Neuinfektionen**

Schätzergebnisse; abhängige Variable: Zahl der COVID-19-Neuinfektionen laut Robert Koch-Institut (RKI)

| | A) Vor den Kontaktbeschränkungen (KW 10-12) | | | | B) Kontakt- und Ausgangsbeschränkungen (KW 14-19) | | | |
|---|---|---|---|---|---|---|---|---|
| Modell | (1) | (2) | (3) | (4) | (5) | (6) | (7) | (8) |
| Neue Fälle im Kreis in der Vorwoche | 1,501***<br>(0,446) | 0,857**<br>(0,361) | 0,857**<br>(0,352) | -0,361<br>(0,259) | 0,595**<br>(0,075) | 0,532***<br>(0,040) | 0,522***<br>(0,043) | 0,501***<br>(0,041) |
| Neue Fälle im Kreis in der Vorwoche x Einwohnerdichte über 1.000 Personen/km² | | 1,092***<br>(0,345) | 0,858**<br>(0,381) | 0,411*<br>0,246) | | 0,139**<br>(0,065) | 0,133**<br>(0,067) | 0,128**<br>(0,064) |
| Neue Fälle im Kreis in der Vorwoche x Bundesland mit Ausgangsbeschränkungen | | | 0,761**<br>(0,316) | 0,763***<br>(0,205) | | | 0,031<br>(0,060) | 0,054<br>(0,043) |
| Neue Fälle im Kreis in der Vorwoche x Zahl der Regentage in der Vorwoche | | | | 0,403***<br>(0,080) | | | | 0,036<br>(0,030) |
| Räumliche Ausbreitung (pendlergewichtet) | 5,755***<br>-1,769 | 5,101***<br>-1,167 | 4,664***<br>(0,918) | 5,030***<br>(0,695) | 0,538***<br>(0,183) | 0,433**<br>(0,201) | 0,422**<br>(0,204) | 0,461***<br>(0,222) |
| Zahl der Regentage in der Vorwoche | | | | -1,443*<br>(0,765) | | | | -1,895*<br>-1,142 |
| Beobachtungszeitraum (Kalenderwochen) | 10-12 | 10-12 | 10-12 | 10-12 | 14-19 | 14-19 | 14-19 | 14-19 |
| Kreis-fixe Effekte | Ja | Ja | Ja | Ja | Ja | Ja | Ja | Ja |
| Kalenderwochen-fixe Effekte | Ja | Ja | Ja | Ja | Ja | Ja | Ja | Ja |
| Angepasstes $R^2$ (volles Modell) | 0,864 | 0,887 | 0,896 | 0,907 | 0,869 | 0,871 | 0,871 | 0,872 |
| Angepasstes $R^2$ (zentriertes Modell) | 0,646 | 0,707 | 0,730 | 0,759 | 0,523 | 0,530 | 0,531 | 0,534 |
| Beobachtungen | 1.203 | 1.203 | 1.203 | 1.203 | 2.406 | 2.406 | 2.406 | 2.406 |

Anmerkungen: Die Standardfehler (in Klammern) sind auf Kreisebene geclustert; ***: $p < 0{,}01$, **: $p < 0{,}05$, *: $p < 0{,}1$. Die Beobachtungseinheiten sind die 401 Kreise und kreisfreie Städte in Deutschland, wobei die Gesamtzahl der COVID-19-Neuinfektionen je Kreis und Kalenderwoche (Meldedatum) aufsummiert wurden. Die abhängige Variable ist die Zahl der Neuinfektionen in der jeweiligen Kalenderwoche und im jeweiligen Kreis. Die erklärenden Variablen beziehen sich jeweils auf die vorangegangene Kalenderwoche.

Quellen: RKI; Statistisches Bundesamt; Statistik der Bundesagentur für Arbeit; eigene Berechnungen.

die Begründung der Maßnahmen Belege in den Daten gibt. Schließlich trug auch eine schlechte Witterung erheblich zu einer erhöhten Ausbreitungsgeschwindigkeit des Virus bei (Modell 4). Gab es in einer Woche zwei Regentage mehr, so steckte eine neu infizierte Person durchschnittlich knapp eine weitere Person innerhalb des Kreises an. Dies deutet darauf hin, dass Kontakte im Freien weniger gefährlich sind als anfangs angenommen wurde, wohingegen Kontakte in geschlossenen Räumen als Hauptübertragungsweg infrage kommen.[3]

In Teil B von Tabelle 1 wurden die Schätzungen aus Teil A für den Zeitraum der Kontakt- und Ausgangsbeschränkungen wiederholt (Kalenderwochen 14 bis 19). Es zeigt sich über alle Schätzungen hinweg, dass die Infektionsdynamik stark abgeschwächt wurde (Modell 5). Zudem waren die Unterschiede zwischen dicht besiedelten und ländlichen Kreisen deutlich kleiner (Modell 6). Statistisch kann kein Unterschied zwischen Bundesländern mit bzw. ohne Ausgangsbeschränkungen mehr nachgewiesen werden (Modell 7). Letzteres zeigt einerseits die Wirksamkeit der ergriffenen Maßnahmen, lässt andererseits allerdings nicht den Schluss zu, dass die strengeren Maßnahmen letztlich unnötig gewesen wären. Auch die Zahl der Regentage hatte in dieser Phase keinen statistisch signifikanten Einfluss auf die Infektionsdynamik, was als Indiz für die Einhaltung der Kontaktbeschränkungen durch weite Teile der Bevölkerung gewertet werden kann, die auch Kontakte in geschlossenen Räumen auf ein Minimum reduzierten (Modell 8). Im Umkehrschluss legt dieses Resultat nahe, dass die in einigen Aprilwochen relativ vollen Parks in Großstädten die Infektionsdynamik nicht wesentlich beschleunigt haben.

## Pendlergeschehen mit dem stärksten Einfluss

In allen Schätzungen ist das Pendlergeschehen von großer Bedeutung für die Verbreitung des Coronavirus – die Intensität der Verflechtung einer Region mit anderen Regionen begünstigt das Infektionsgeschehen mit einer gewissen zeitlichen Verzögerung. Kommt es in einem Landkreis zu

3 Der Interaktionsterm der Zahl der Regentage ist auch in Schätzungen mit weniger Interaktionstermen hoch signifikant. Der schwach signifikante Haupteffekt ist weniger robust und in anderen Spezifikationen teils insignifikant.

Abbildung 2
**Aufenthaltsorte nach Mobilfunkzellen**
Abweichungen in % gegenüber dem jeweiligen Durchschnitt einer Woche

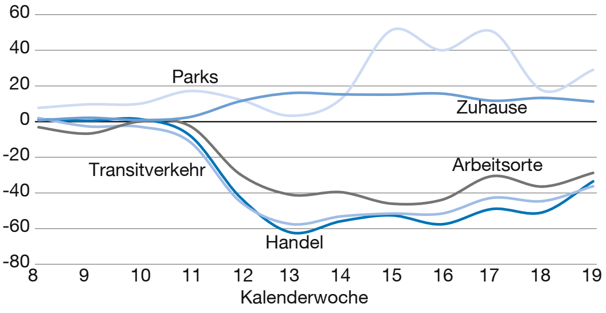

Quelle: Google; eigene Berechnungen.

Abbildung 3
**Zerlegung der Modelleffekte: Vorhersage des Modells und tatsächliche Zahl der gemeldeten Fälle**

Modellprognose, basierend auf den Daten der Vorwoche

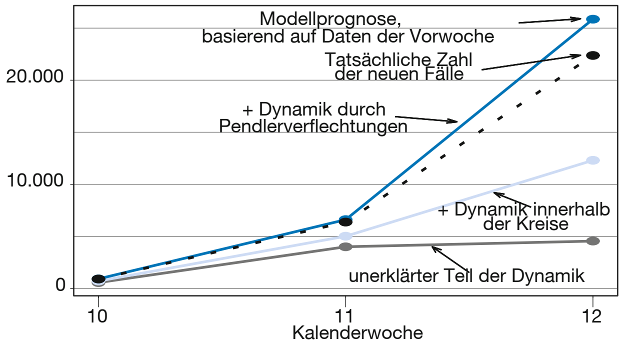

Quelle: Robert Koch-Institut; eigene Berechnungen.

vermehrten Corona-Fällen, ist dies in der folgenden Woche in eng verflochtenen anderen Regionen ebenfalls der Fall.

Dieser Zusammenhang ist nach den weitreichenden Maßnahmen ab der 12. Kalenderwoche zwar weiterhin statistisch signifikant, jedoch quantitativ wesentlich weniger relevant (vgl. Tabelle 1, Teil B). Offenbar wurden die Pendelbewegungen und Verflechtungen der Regionen erheblich geringer (vgl. Abbildung 2). Dies zeigen auch Auswertungen zur Mobilität. Die Verbreitung des Virus im Raum kann daher vor allem durch geringere Pendelbewegungen eingeschränkt werden. Diese Beobachtung steht im Einklang mit Untersuchungen zur Verbreitung der saisonalen Grippe in Frankreich (Adda, 2016). Um die Bedeutung der Pendlerverflechtungen relativ zu anderen Einflussfaktoren schätzen zu können, zeigt Abbildung 3 eine Zerlegung der geschätzten Zahl der Neuinfektionen in den Kalenderwochen 10 bis 12. Die Vorhersagen basieren jeweils auf den gemeldeten neuen Fällen aus der Vorwoche und wurden mittels Modell (2) aus Tabelle 1 erstellt.

Es zeigt sich deutlich, dass die Pendlerverflechtungen großen Einfluss auf die Infektionsdynamik in Deutschland hatten. Insbesondere in Kalenderwoche 12 sind die mittelbar durch Pendlerverflechtungen entstehenden Infektionen für den mit Abstand größten Teil der neuen Fälle verantwortlich. Das liegt daran, dass in Kalenderwoche 11 bereits in fast allen Teilen Deutschlands Infektionen mit COVID-19 gemeldet wurden, sodass es in der Folgewoche immense Wechselwirkungen zwischen den Kreisen geben konnte. Auch in Kalenderwoche 11 ist der Anteil der Pendlerverflechtungen bereits höher als der Anteil der Dynamik innerhalb des Kreises.[4] Die wochen- und kreis-fixen Effekte können vom Modell nicht explizit erklärt werden. Sie fangen durchschnittliche Niveauunterschiede zwischen den Kreisen und zwischen den verschiedenen Wochen ab, wie beispielsweise Unterschiede im Testverhalten der Bevölkerung und der Gesundheitsämter, sowie Änderungen der nationalen Testkapazitäten.

## Kontaktbeschränkungen wirken schnell

Um besser bewerten zu können, wie schnell die Kontaktbeschränkungen sowie die zahlreichen begleitenden Maßnahmen zu einer Eindämmung der Ausbreitung von COVID-19 beigetragen haben, wurde das Basismodell mit einer reduzierten Zahl an Regressionstermen separat für jede Kalenderwoche geschätzt. Abbildung 4 zeigt die Ergebnisse dieser Schätzungen für die Kalenderwochen 10 bis 19. Eine markante Beobachtung ist, dass mit der Einführung weitgehender Beschränkungen in der 12. Kalenderwoche die Unterscheide zwischen den Regionstypen verschwinden. Dieser Trend der Angleichung setzte bereits vor den eigentlichen Maßnahmen ein: Möglich scheint, dass Menschen ihr Verhalten bereits vorher angepasst haben und die Fernsehansprache von Bundeskanzlerin Angela Merkel das Gefahrenbewusstsein erhöht hat. Erste Ergebnisse zu den Effekten offizieller Ansprachen deuten zumindest darauf hin, dass diese signifikanten Einfluss auf die Erwartungsbildung in der Bevölkerung haben (Haan et al., 2020).

## Fazit: Homeoffice stärken – Freiräume nutzen

Ein wichtiger Verbreitungsweg des Coronavirus scheint über die interregionalen Pendlerverflechtungen zu führen. Darauf deuten die vorgelegten Auswertungen hin, die in Einklang mit Ergebnissen anderer Studien zur Verbreitung der saisonalen Grippe stehen. Pendlerbewegungen können einerseits durch Wege zur Arbeit entstehen – andererseits durch Fahrten an zentrale Orte mit entsprechenden Einzelhandels- und Dienstleistungsangeboten. Allerdings unter-

4 Es ist anzumerken, dass das Modell die Dynamik in Kalenderwoche 12 leicht überschätzt.

Abbildung 4

**COVID-19: Infektionsgeschehen nach Bevölkerungsdichte und räumliche Spillover**

Zahl Neuinfizierter in Relation zu den Infektionen der Vorwoche

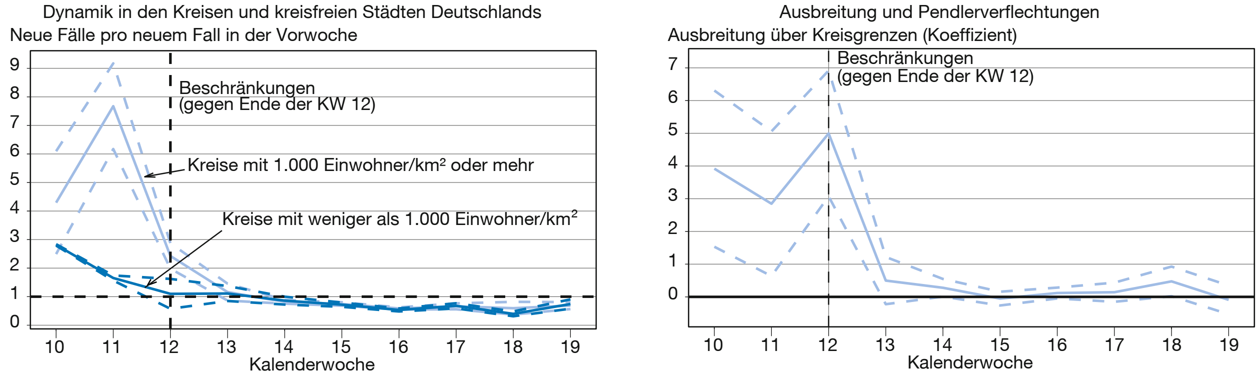

Quelle: Robert Koch-Institut; eigene Berechnungen.

scheiden sich Arbeitskontakte oft deutlich von flüchtigeren Kontakten beim Einkaufen oder vergleichbaren Aktivitäten. So sitzen Menschen in Großraum- und Gemeinschaftsbüros in der Regel viele Stunden nebeneinander, während Aufenthalte in Geschäften in vielen Fällen nur wenige Minuten dauern. Außerdem finden Pendlerbewegungen an den Arbeitsort wesentlich häufiger statt als Besuche in der Stadt zum Einkaufen. Lockerungen sollten diese beiden Faktoren getrennt voneinander betrachten: So ist es für den Einzelhandel und für Dienstleistungsunternehmen wichtig, ihren Geschäftsbetrieb wieder aufnehmen zu können. Dies erzeugt zwar ebenfalls intensivere Pendlerbewegungen, was die Verbreitung des Virus begünstigt. Dem kann aber durch Hygienemaßnahmen entgegengewirkt werden. Andererseits können die Möglichkeiten des Homeoffice verstärkt genutzt werden, ohne große ökonomische Kosten zu verursachen. Dabei reduziert sich vor allem die Frequenz, mit der sich Menschen aus verschiedenen Wohnorten begegnen, was die Ausbreitungsdynamik stark abbremsen kann. Dies ist offenbar ein sehr wirksamer Weg, um das Infektionsgeschehen zu reduzieren.

In den vergangenen Wochen haben bereits viele Unternehmen von dieser Chance Gebrauch gemacht. Dies auch politisch zu unterstützen, wäre eine Möglichkeit, das Epidemiegeschehen einzugrenzen. Eine Möglichkeit wäre, die Anschaffung notwendiger Infrastruktur für ein effizientes Homeoffice zu unterstützen und die Einrichtung von Heimarbeitsplätzen entweder durch Zuschüsse oder besondere steuerliche Abschreibungsmöglichkeiten attraktiver zu gestalten. Es fehlen weiterhin stichhaltige Belege für die relative Wichtigkeit verschiedener Übertragungswege. Diese wären jedoch sehr wichtig, damit Maßnahmen stärker hinsichtlich ihres Kosten-Nutzen-Profils bewertet werden können. Ein langfristig tragbares Bündel aus Maßnahmen muss sowohl ökonomisch vertretbar als auch wirkungsvoll sein, um den nötigen Rückhalt in der Bevölkerung zu haben.
